# Effects of Nitrogen Addition on Nitrogen Resorption in Temperate Shrublands in Northern China

**DOI:** 10.1371/journal.pone.0130434

**Published:** 2015-06-18

**Authors:** Jianhua Zhang, He Li, Haihua Shen, Yahan Chen, Jingyun Fang, Zhiyao Tang

**Affiliations:** 1 State Key Laboratory of Vegetation and Environmental Change, Institute of Botany, Chinese Academy of Sciences, Beijing, China; 2 University of Chinese Academy of Sciences, Beijing, China; 3 Department of Ecology, College of Urban and Environmental Sciences, and Key Lab for Earth Surface Processes of the Ministry of Education, Peking University, Beijing, China; Shandong University, CHINA

## Abstract

Nutrient resorption from senescing leaves is a key mechanism of nutrient conservation for plants. The nutrient resorption efficiency is highly dependent on leaf nutrient status, species identity and soil nutrient availability. Nitrogen is a limiting nutrient in most ecosystems, it is widely reported that nitrogen resorption efficiency (NRE) was highly dependent on the soil nitrogen availability and vary with N deposition. The effects of nitrogen deposition on NRE and nitrogen concentration in green and senescing leaves have been well established for forests and grasslands; in contrast, little is known on how plants in shrublands respond to nitrogen deposition across the world. In this study, we conducted a two-year nitrogen addition manipulation experiment to explore the responses of nitrogen concentration in green and senescing leaves, and NRE of seven dominant species, namely, *Vitex negundo*, *Wikstroemia chamaedaphne*, *Carex rigescens* and *Cleistogenes chinensis* from the *Vitex negundo* community, and *Spirea trilobata*, *Armeniaca sibirica*, *V*. *negundo*, *C*. *rigescens* and *Spodiopogon sibiricus* from the *Spirea trilobata* community, to nitrogen deposition in two typical shrub communities of Mt. Dongling in northern China. Results showed that NRE varied remarkably among different life forms, which was lowest in shrubs, highest in grasses, and intermediate in forbs, implying that shrubs may be most capable of obtaining nitrogen from soil, grasses may conserve more nitrogen by absorption from senescing leaves, whereas forbs may adopt both mechanisms to compete for limited nitrogen supply from the habitats. As the N addition rate increases, N concentration in senescing leaves ([N]_s_) increased consistent from all species from both communities, that in green leaves ([N]_g_) increased for all species from the *Vitex negundo* community, while no significant responses were found for all species from the *Spirea trilobata* community; NRE decreased for all species except *A*. *sibirica* from the *Vitex* community and *W*. *chamaedaphn* from the *Spirea* community. Given the substantial interspecific variations in nutrient concentration, resorption and the potentially changing community composition, and the increased soil nutrient availability due to fertilization may indirectly impact nutrient cycling in this region.

## Introduction

Nutrient resorption from senescing leaves is an important mechanism for plants to conserve nutrient in infertile environments. As an important physiological process for nutrient retention in terrestrial plants, nutrient resorption reduces the nutrient loss in litter dropped on the floor and minimizes the dependence of plants on soil nutrient availability [1–3]; the nutrient resorption increases the residence time of nutrients within plants and enables a quick recycling of nutrients through plants and environments [4–7]. In addition, nutrient resorption from senescing plant tissues affects litter quality and litter decomposition, and consequently influences nutrient cycling [1,8]. It is widely reported that the percentage of nutrient absorbed during senesce, defined as nutrient resorption efficiency (NuRE), is highly dependent on leaf nutrient status at the global scale and varies remarkably among species and sites [7,9–12]. It has also been proposed that NuRE decreased with nutrient availability, and plants from low-nutrient habitats absorbed more nutrients from the senescing leaves [8,13–16].

As a limiting element in many ecosystems, approximately 50%, on average, of the leaf N is recycled via resorption, varying among plant functional groups and sites [3,4,12,17]. Previous studies have shown a decrease in nitrogen resorption efficiency (NRE) with increases in soil N availability [3,9,15,18], albeit non-significant relationship between NRE and soil N availability has also been reported in some other studies [4,5,19].

The past several decades have witnessed a consistent increase of soil N availability as a result of N deposition caused by the direct fertilization and the increase in N mineralization and nitrification [20–22]. One can reasonably predict that the increasing soil N availability may decrease the NRE of plants. Indeed, such decreases of NRE with soil N availability have been extensively reported in forest and grassland ecosystems [3,18,23–26]. By contrast, little is known about the response of shrubs to N addition [27].

Compared to forests, the natural or semi-natural shrub communities are mostly distributed in nutrient poor sites and the plant growth is severely limited by soil nutrient availability [28,29]. Such nutrient- poor ecosystems are usually considered relatively vulnerable to environmental changes such as N deposition and climate change [20,30–32]. Increased N input to ecosystems may remarkably influence plant nutrient resorption in shrublands. Plants in these ecosystems strongly depend on internal nutrient cycling and are thought to have relatively high NuRE [3].Thus, understanding responses of leaf nutrient resorption to increasing soil nutrient availability is crucial for exploring plant nutrient conservation strategies and nutrient cycling in the shrublands. In this study, we conducted a two-year nitrogen addition manipulation experiment to explore the responses of nitrogen concentration in green and senescing leaves, and NRE of dominant species to nitrogen deposition in two typical shrub communities of Mt. Dongling in northern China. We hypothesize that (1) nutrient additions decrease NRE and nitrogen resorption proficiency (NRP). As deep-rooted plants may mainly rely on acquiring N from soil rather than from senescing leaf to adapt to the nutrient-poor habitats [33], we further hypothesize that (2) shrubs (deep-rooted) may contain higher [N]_s_ (lower NRP) and lower NRE than perennial herbaceous plants (shallow-rooted).

## Materials and Methods

### Ethics Statement

Institute of Botany, Chinese Academy of Sciences has had a permit from local forestry authorities (Beijing forestry bureau, http://www.greenbeijing.gov.cn/) to conduct the experiments in each location. This research was carried out in compliance with the laws of People’s Republic of China. The field studies did not involve endangered or protected species.

### Study site

The experiments were conducted in two typical shrubland communities, the *Vitex negundo* community (*Vitex* community hereafter) and the *Spirea trilobata* community (*Spirea* community hereafter), with general information shown in Table 1, on Mt. Dongling (39°48′N –40°02′N in latitude, 115°24′E –115°36′E in longitude, with a peak of 2303 m) in Beijing, northern China. The area is characterized by a temperate semi- humid climate with a mean annual temperature (MAT) of 6.3°C and a mean annual precipitation of 612 mm. From lowland to the summit, the vegetation changes from shrubland dominated by *V*. *negundo* and *S*. *trilobata* (400–1100 m), deciduous broad- leaved forest dominated by *Quercus liaotungensis* and coniferous forest dominated by *Pinus tabulaeformis* (1100–1700 m), to subalpine meadow (1700–2300 m) [34]. The study area has rarely been polluted, with the background N deposition of 15kg N ha^-1^yr^-1^ [35].

**Table 1:** Elevation, mean annual temperature (MAT) and soil properties of the experimental sites.

Community type	Elevation	MAT	pH	STN	STC	STP
	(m)	(°C)		(mg.g^-1^)	(mg.g^-1^)	(mg.g^-1^)
***Vitex negundo***	791	8.2	8.7	2.52^a^(0.33)	26.36^a^(4.38)	0.50^a^(0.05)
***Spiraea trilobata***	1170	6.4	8.9	2.20^a^(0.24)	34.71^a^(5.49)	0.50^a^(0.02)

STN = soil total N; STC = soil total C; STP = soil total P. Values of STN, STC, STP are expressed as mean with standard error (SE) in the parentheses of three samples.

^a^ Values with the same letter in a column are not significantly different (Turkey multiple comparison test; *p*> 0.05).

### Experiment design

In each community, twelve 5×5 m^2^ plots with approximately similar stand density were established based on a four treatments and three replicates random block design. The treatments included four levels of N addition: control (0 kg N ha^-1^yr^-1^), low-N (20 kg N ha^-1^yr^-1^), medium-N (50 kg N ha^-1^yr^-1^) and high-N (100 kg N ha^-1^yr^-1^). We used CO(NH_2_)_2_ for the N addition. Fertilizer additions were divided into five equal monthly doses (May–September) since May, 2012. In each month, the CO(NH_2_)_2_ was dissolved with two liters of water, and sprayed under the canopy using a backpack sprayer. The Control plots received two liters of water without N addition.

### Field sampling and analyses

In each plot, only the central area of 4.5 × 4.5 m^2^ was used for leaf sampling. The sampling area was further divided into nine 1.5 × 1.5 m^2^ subplots. We selected seven dominant perennial species, including four from the *Vitex* community (*V*. *negundo*, *W*. *chamaedaphne*, *Carex rigescens* and *Cleistogenes chinensis*), and five from the *Spirea* community (*S*. *trilobata*, *V*. *negundo*, *Carex rigescens*, *A*. *sibirica*, and *S*. *sibiricus*). These species included two grasses (*Cleistogenes chinensis*, *S*. *sibiricus*), one sedge (*Carex rigescens*) and four deciduous shrubs (*W*. *chamaedaphne*, *V*. *negundo*, *S*. *trilobata* and *A*. *sibirica*). Following Cornelissen et al. (2003) [36], we randomly selected and marked three healthy individuals for each woody species in each plot. We collected more than 30 fully expanded sun leaves during the growing season (early August) and collected freshly senesced leaves directly from the corresponding individuals in the falling season (October–November), while the same number of leaves for each of herbaceous species were randomly collected within each plot. At each site, we collected three soil samples at 0–20 cm depth in each plot. All soil samples were sieved through a 100-mesh sieve to determine soil inorganic N (SIN, NO3^–^-N and NH4^+^-N), total phosphorus (STP), total carbon (STC), total N (STN) concentrations and soil pH. Leaf samples were dried at 65°C for 24–48 h to constant weight and then were ground with a ball mill (NM200, Retsch, Haan, Germany).

Leaf [N], STN and STC were measured using a 2400 IICHNS/O Elemental Analyzer (Perkin-Elmer, USA), and STP was measured by a molybdate/ascorbic acid method after H_2_SO_4_-HClO_4_ digestion [37]. SIN was measured with a continuous flow spectrophotometer (FIAstar 5000; Foss Tecator, Denmark) using the fresh soil samples extracted with 50 ml of 2 M KCl [38]. The air-dried soil samples were extracted with distilled water without carbon dioxide (1:5 v/v) to measure the pH.

The data which forms the basis for the analysis can be found in S1 Dataset and S2 Dataset and S3 Dataset.

### Calculation of N resorption efficiency (NRE)

NRE was defined as the percentage of N absorbed during senesce, and calculated as the ratio of difference in [N]_g_ and senescent leaves [N]_s_ to [N]_g_ [39]:

NRE=([N]g−[N]s)[N]g×100% (1)

In addition, the [N]_s_ was also used as an indicator of NRP, as it represents the level to which nutrient is reduced during leaf senescence.

### Statistical analysis

We first calculated the means of [N]_g_, [N]_s_ and NRE for each species under different N treatments. The effects of treatment on leaf [N] and NRE were examined for each species individually based on one-way ANOVAs with *LSD* test [40]. Three-way ANOVA was used to test the main and interactive effects of N addition, species identity and community type on [N]_g_, [N]_s_ and NRE. Statistical analysis was conducted by the SPSS version17.0 [40].

## Results

**Fig 1:**
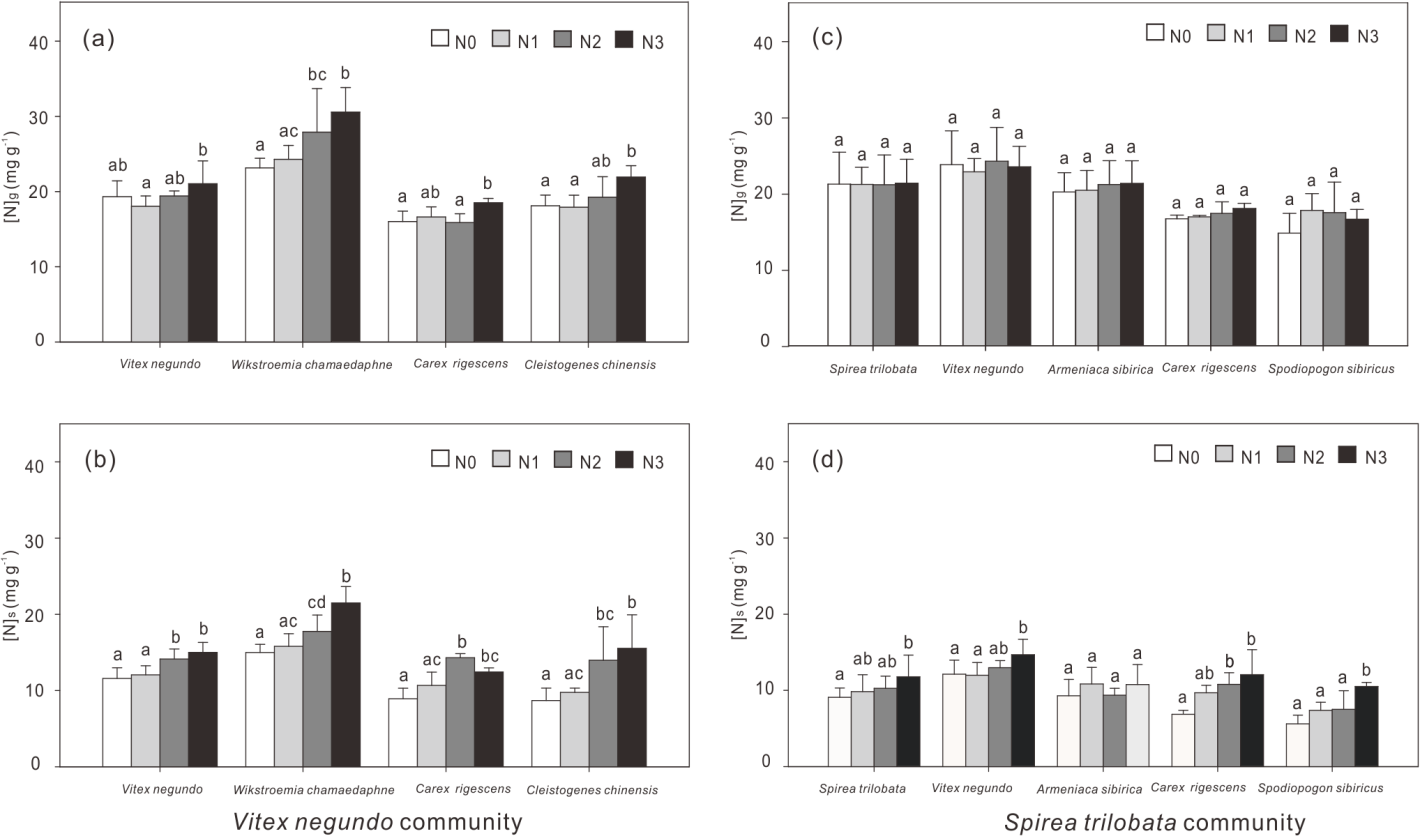
Responses of mean N concentrations in green ([N]_g_) and senescing leaves ([N]_s_) to N addition for seven dominant species in the *Vitex negundo* (left, a and b) and *Spirea trilobata* (right, c and d) communities. Error bars indicate standard deviation (n = 6). Different letters on the error bar indicate significant differences among treatments for each species based on one-way ANOVA.

N addition rate, species identity and community type significantly affected the [N]_g_ (P<0.01) (Table 2). [N]_g_ responded to N addition in different way between these two communities. In the *Vitex* community, [N]_g_ increased with N addition rate for all species (Fig 1A); In the *Spirea* community, [N]_g_ also exhibited similar trends with N addition across all species, although not significant (Fig 1C).

**Table 2:** Effects of species, community type and N addition rate and their interaction on N concentrations in green ([N]_g_) and senescent leaves ([N]_s_) and N resorption efficiency (NRE) in the shrubland communities of Mt. Dongling.

		[N]_g_			[N]_s_			NRE		
	df	MS	F	*p*	MS	F	*p*	MS	F	*p*
**N**	3	48.1	7.5	**<0.001**	129.2	35.5	**<0.001**	1440.3	15.4	**<0.001**
**Sp**	6	202.3	31.4	**<0.001**	100.3	27.6	**<0.001**	334.8	3.6	**0.002**
**Ct**	1	74.8	11.6	**0.001**	8.0	2.2	0.140	1521.5	16.3	**<0.001**
**N×Sp**	18	5.7	0.9	0.606	4.8	1.3	0.170	123.5	1.3	0.179
**N×Ct**	3	3.2	0.5	0.680	1.5	0.4	0.740	84.0	0.9	0.443
**Sp×Ct**	1	46.8	7.3	**0.008**	4.8	1.3	0.250	44.9	0.5	0.489
**N×Sp×Ct**	2	0.7	0.1	0.901	1.9	0.5	0.600	75.9	0.8	0.445

N, N addition treatment; Sp, Species; Ct, Community type.

Both N addition rate and species identity affected the [N]_s_ of the seven species (P<0.001) (Table 2). [N]_s_ was highest in the shrubs and lowest in grasses, and it increased with the N addition rate for all species, except *A*. *sibirica* (Fig 1B and 1D).

**Fig 2:**
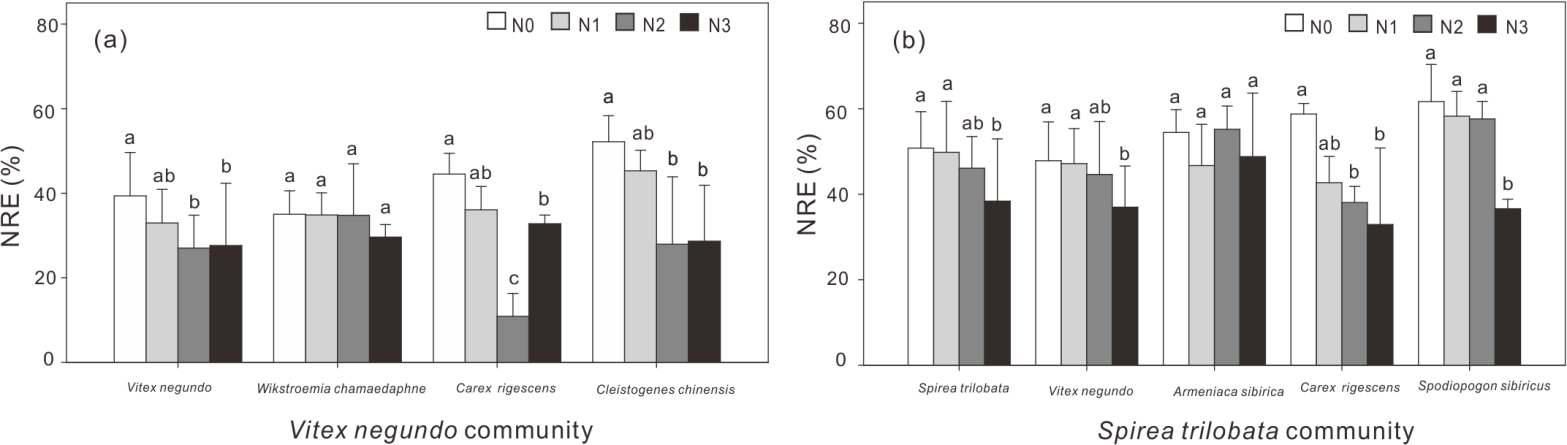
Responses of mean N resorption efficiency (NRE) to N addition for seven dominant species in the *Vitex negundo* (a) and *Spirea trilobata* (b) communities. Error bars indicate standard deviation (n = 6). Different letters on the error bars indicate significant differences among treatments for each species based on one-way ANOVA. Error bars indicate standard deviation (n = 6). Different letters on the error bars indicate significant differences among treatments for each species based on one-way ANOVA.

ANOVA illustrated that N addition rate (P<0.001), species identity (P = 0.002) and community type (P<0.001) significantly affected NRE (Table 2). For all species except *A*. *sibirica* and *W*. *chamaedaphne*, NRE decreased with N addition (Fig 2A and 2B).

**Fig 3:**
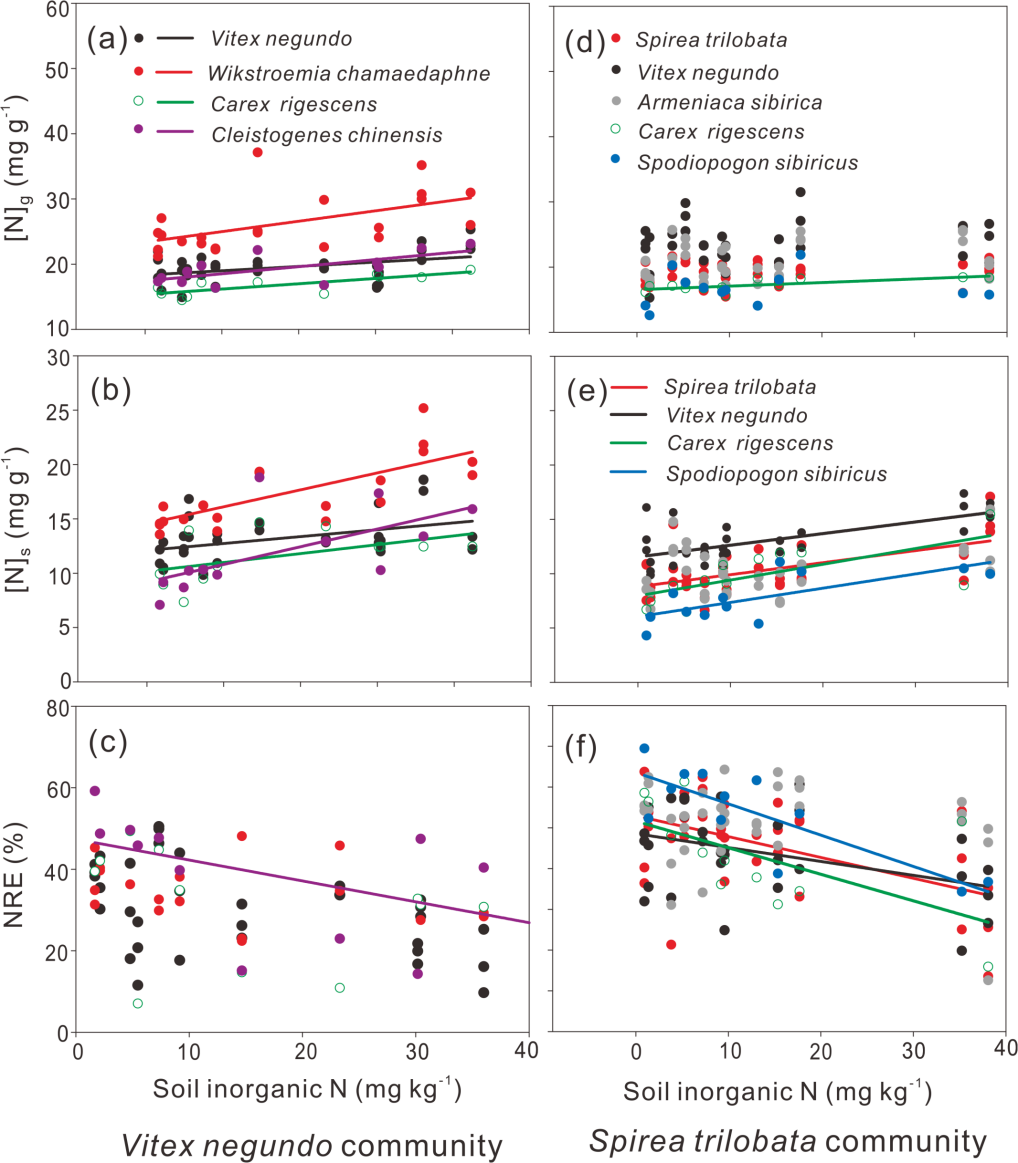
Changes of N concentration in green ([N]_g_), senescing leaves ([N]_s_) and nitrogen resorption efficiency (NRE) with soil inorganic N in the *Vitex negundo* (left, a, b and c) and *Spirea trilobata* (right, d, e and f) communities. The solid lines indicate significant linear regression at p< 0.05.

[N]_g_ increased with SIN for all species from the *Vitex* community and *C*. *rigescens* from the *Spirea* community (Fig 3A and 3D); [N]_s_ increased with SIN for all species from the *Vitex* community and those from the *Spirea* community (Fig 3B and 3D). NRE decreased with SIN for *C*. *chinensis* from the *Vitex* communtiy and *S*. *trilobata*, *V*. *negundo*, *C*. *rigescens* and *S*. *sibiricus* from the *Spirea* community (Fig 3C and 3F).

## Discussion

### Influence of nitrogen addition on the nitrogen resorption traits

Consistent with other experimental studies in grasslands [14,18] and forests [41], we found a decrease of NRE in response to N addition for all species except *A*. *sibirica* and *W*. *chamaedaphne* (Figs 1 and 2). Such a decrease in NRE is probably resulted from the increase of [N]_g_ with increased N supply from the soil [9,42], implying that plants will absorb more N from soils and become less dependent on N resorbed from senescing leaves.

Some studies alleged that [N]_s_ was more responsive to changes in soil N availability because it is not subject to temporal variation in [N]_g_ and timing of sampling [5,9,16,25], and further proposed to use NRP as an indicator for potential of nutrient resorption [5,25,43]. In this study, we observed inconsistent response of [N]_g_ and NRE, but a consistent increasing [N]_s_ to soil N availability across species (Fig 3), suggesting that NRP (or [N]_s_) can be applied to illustrate the status of nutrient resorption.

**Fig 4:**
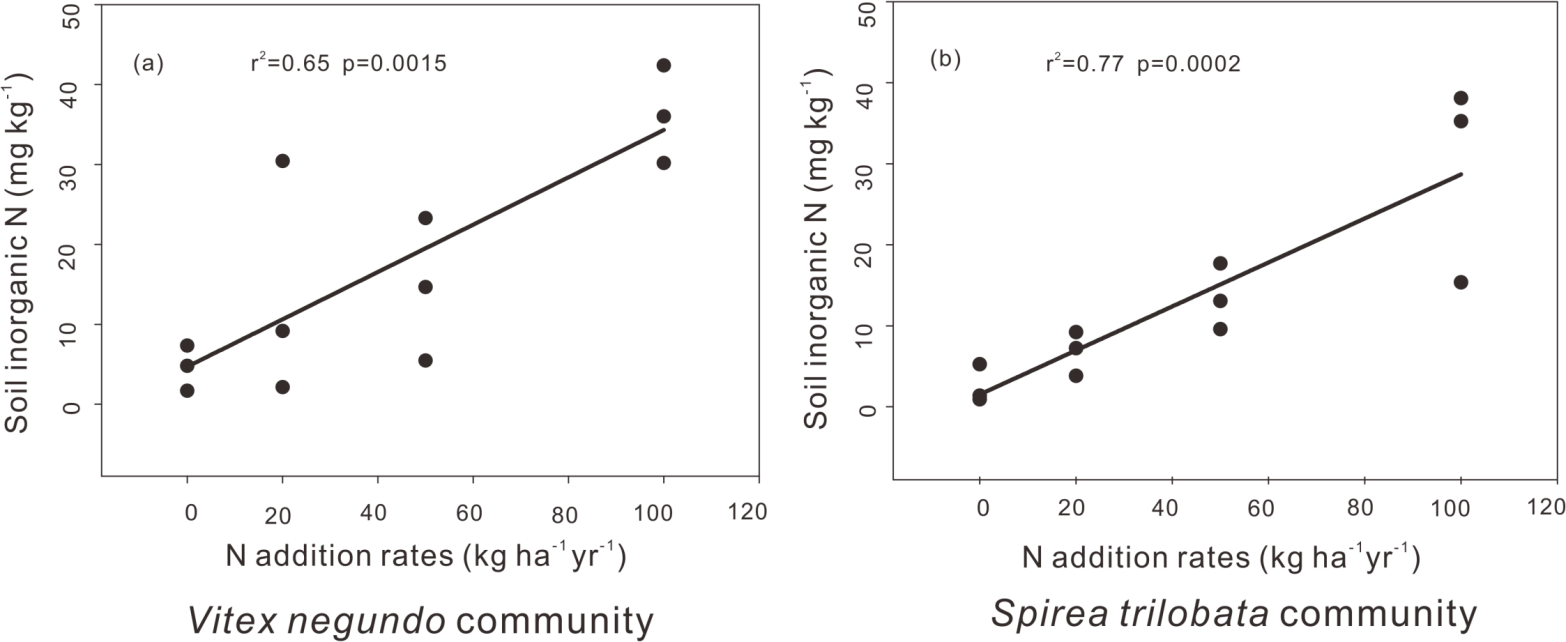
Relationships between soil inorganic N and N addition rates in the *Vitex negundo* (a) and *Spirea trilobata* communities. The solid lines indicate significant linear regression at p< 0.05.

It has been widely acknowledged that NuRE may be determined by the relative cost the plants absorbing nutrient from the senescing leaves versus from soil, but not the absolute levels of soil nutrient [7,16]. In nutrient rich soils, the energy cost for plants to absorb nutrients from soils was lower than that from senescing leaves [16]. In our study, we found increased soil inorganic N (Fig 4), but decreased nitrogen resorption efficiency, with N addition rate, supporting the idea that N addition influence nitrogen resorption efficiency through soil nitrogen availability.

### Species and life form dependent response of N resorption to N addition

Consistent with previous studies [3,4,24], we observed species-specific response of leaf chemistry to N addition, as NRE of two shrubs (*A*. *sibirica* and *W*. *chamaedaphne*) did not change with N addition (Fig 2). The species-specific response of N resorption traits to N addition was more apparent in [N]_g_, as five species from the *Spirea* community did not show a consistent increase in [N]_g_ with N addition (Fig 1). Such species-specific response can be confirmed by the significant effects of species and the interaction between species and nitrogen addition rate on [N]_g_, [N]_s_ and NRE detected by ANOVA (Table 2).

Previous experimental studies on the nutrient resorption in forests focus mainly on the dominant tree species [26], hardly compared the difference in responses of nutrient resorption of species from different layers. Therefore, knowledge on nutrient conservation strategies of different life forms in very limited. Thus, in this study, we compared the NRE of shrubs, grasses and forbs of shrublands. We found highest NRE in grasses but lowest in shrubs (Fig 2), implying that the grasses conserve N via resorption from senescing leaves rather than via absorption N in green leaves, while shrubs prefer to uptake N rather than resorb N via senescing leaves. The results were different from Yuan et al. (2005) [44] (NRE: herbs> shrubs> trees> graminoids) and Jiang et al. (2012) [31] (NRE: sedges > grasses), but consistent with Lü et al. (2011) [45] (NRE: grasses > forbs > shrubs). We further observed different responses of NRE to soil inorganic N among life forms, with the herbaceous plants (grasses and forbs) were more sensitive than shrubs to soil inorganic N (Fig 3), which might be partly explained by the fact that herbaceous species are more efficient in N resorption than other life-forms [3]. Shallow-rooted plants in the shrubland ecosystems, such as perennial grasses, may highly depend on internal nutrient cycling and are considered to contain relatively high NuRE [3,16] to fulfill the nutrient requirement for high leaf area index, and fast growth and tissue uptake [46]. On the contrary, the deep rooted shrubs may adapt to the inferitle habitat through uptaking nutrient from the soil [33].

[N]_g_ reflects the ability of plant species to acquire N on one hand, and the ability to conserve and utilize N on the other hand, with low [N]_g_ is considered an efficient mechanism for N utilization and conservation [9,39,47]. Based on these arguments, we proposed that shrubs are most capable of acquiring N, whereas grasses conserve more N by absorbing less N in leaves, while forbs adopt both methods in their competition for limited N supply from their habitats.

### Implications for ecosystem N cycling

[N]_s_ increased, while NRE decreased, with N addition for the nearly all species, indicating that communities in N-poor inhabits may resorb more N from the senescing leaves and are more N-resorption dependent. The increasing [N]_s_ with N addition indicated higher litter N contents in the communities, and therefore increased the amount of N returned to the soil via leaf litter production [25,41]. Moreover, leaf litter decomposition is mainly controlled by N concentration in the litter [48,49], with N-high litter being more easily decomposed [11,49], thus N addition may lead to more rapid N returns to soils. It has been reported that increased N availability can affect N cycling via shifts in species composition of vegetation [23,50,51]. Our study implies that species-specific responses of leaf nutrient resorption to N addition may largely affect plant-mediated nutrient cycling, and then ecosystem structure and functioning in the shrublands.

## Conclusions

In summary, nitrogen resorption efficiency differed remarkably among life-forms in the temperate shrublands of Mt. Dongling in northern China. It was highest in grasses but lowest in shrubs. These results indicated that shrubs may be most capable of acquiring nitrogen from soil, and grasses may conserve more nitrogen by absorption from senescing leaves, while the forbs may adopt both mechanisms, in their competition for limited nitrogen supply from the habitats. Two-year nitrogen addition increased the nitrogen concentration in the senescing leaves but decreased nitrogen resorption efficiency in the temperate shrublands of Mt. Dongling in northern China. Nitrogen concentration in green leaves exhibited species specific response to nitrogen addition. The species-specific responses of leaf nutrient resorption to nitrogen addition can largely affect plant-mediated nutrient cycling in this region.

## Supporting Information

S1 Dataset Leaf nitrogen concentration under different fertilization treatments in the *Vitex negundo* community. (CSV)

S2 Dataset Leaf nitrogen concentration under different fertilization treatments in the *Spireae trilobata* community. (CSV)

S3 Dataset Soil properties under different fertilization treatments in the *Vitex negundo* and *Spireae trilobata* communities. (CSV)
